# Supplementary material for: High temperatures alter cross-over distribution and induce male meiotic restitution in Arabidopsis thaliana
Source: Commun Biol. 2020 Apr 23;3:187. doi: 10.1038/s42003-020-0897-1 (PMC7181631; doi:10.1038/s42003-020-0897-1)
Supplement: Supplementary file 1 — Supplementary Information [file 42003_2020_897_MOESM1_ESM.pdf]

SUPPLEMENTARY FIGURES – Arabidopsis male meiosis under heat

De Storme Nico & Geelen Danny

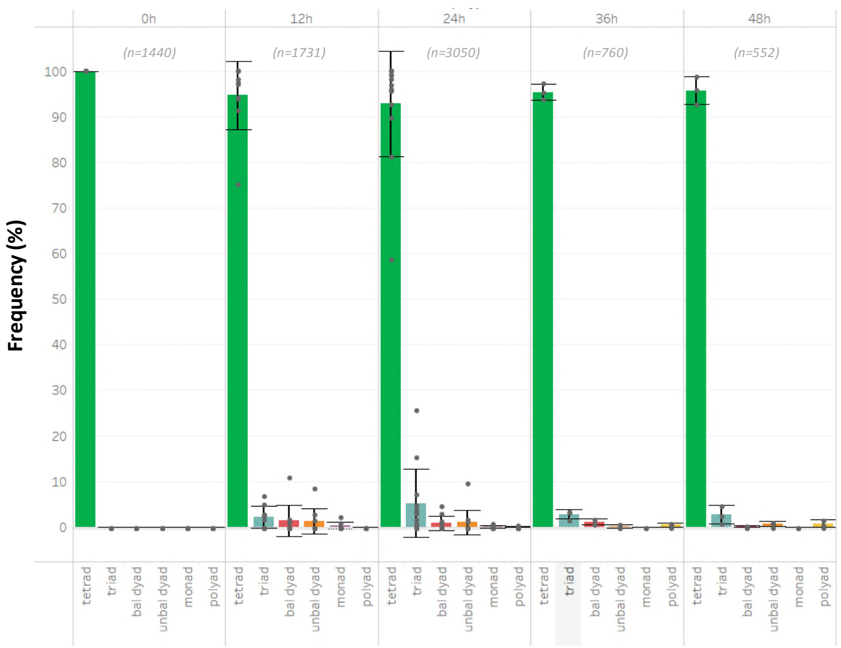

Supplementary Fig. 1. Quantitative analysis of the different types of male meiotic figures produced by *Arabidopsis thaliana* 2x Col-0 male sporogenesis exposed to varying periods of mild heat stress (26-28°C). Presented values represent the mean of at least 500 male meiotic products isolated from at least three different plants. The total number of meiotic products analysed for each specific treatment is indicated by value n. Error bars represent standard deviation values.

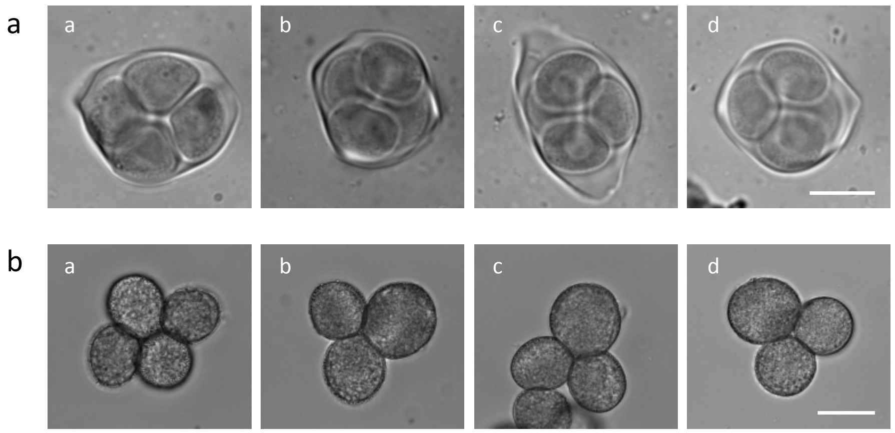

Supplementary Fig. 2. (a) Tetrad-stage male meiocytes of *Arabidopsis thaliana* Col-0 diploid plants under normal temperature conditions (a) and 12 hours post heat shock treatment for 24h at 30-32°C (b-d). For both treatments, at least three different plants were assessed and a minimum number of 100 meiocytes were assessed. Scale bar, 10 µm. (b) Mature pollen configurations in the *qrt1-2*$^{-/-}$ Arabidopsis

Col-0 diploid background showing tetrad figures under normal temperature conditions (18-20°C; a) and meiotically restituted dyads and triads at 7 days following heat shock treatment for 24h at 30-32°C (b-d). For both treatments, at least three different plants were assessed and a minimum number of 100 meiocytes were assessed. Scale bar, 20 µm.

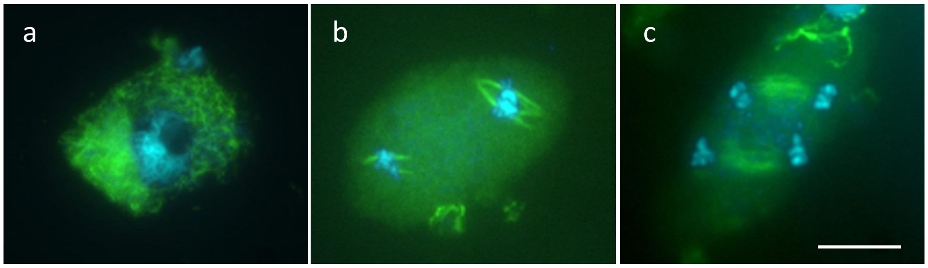

Supplementary Fig. 3. Tubulin α immunostaining of DAPI-stained chromosome spreads of Arabidopsis Col-0 male meiocytes at prophase I (a), metaphase II (b) and anaphase II (c) under conditions of heat stress (24h 30-32°). DAPI-stained chromosomes are indicated in blue, cytoskeletal microtubule figures labeled with α-tubulin antibody are indicated in green. Scale bar, 10 µm.

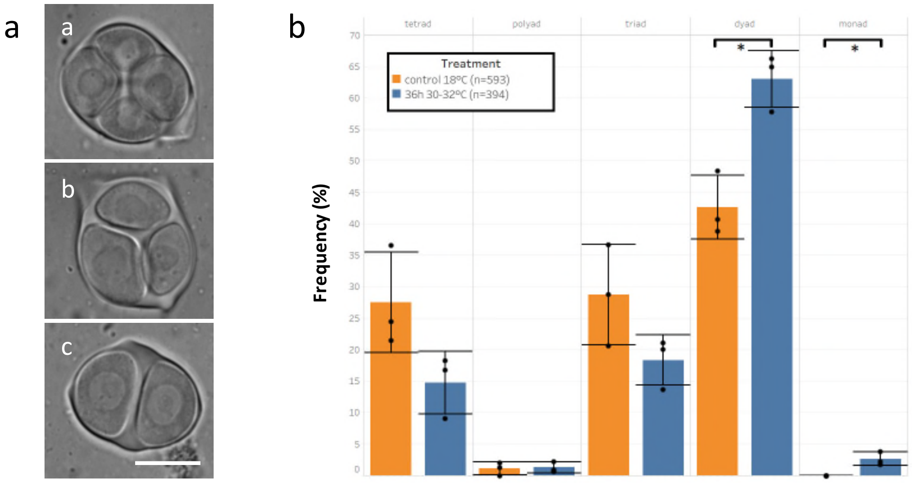

Supplementary Fig. 4. (a) Representative images of a balanced tetrad (a), triad (b) and balanced dyad (c) produced by *atps1-1*$^{-/-}$ Arabidopsis male meiosis under normal temperature conditions (18°C) . Scale bar, 10 µm. (b) Frequency of different types of male meiotic products generated by the *atps1-1*$^{-/-}$ Arabidopsis mutant under normal temperature conditions (18°C) and following heat shock treatment (36h at 30-32°C). Statistical differences in the frequency of each type of meiotic product between control and heat-stressed *atps1*$^{-/-}$ male sporogenesis are assessed via a t-test (α = 0,05) and are indicated by asterisk labels above the corresponding bar plots. For both treatments, three independent biological repeats were performed.

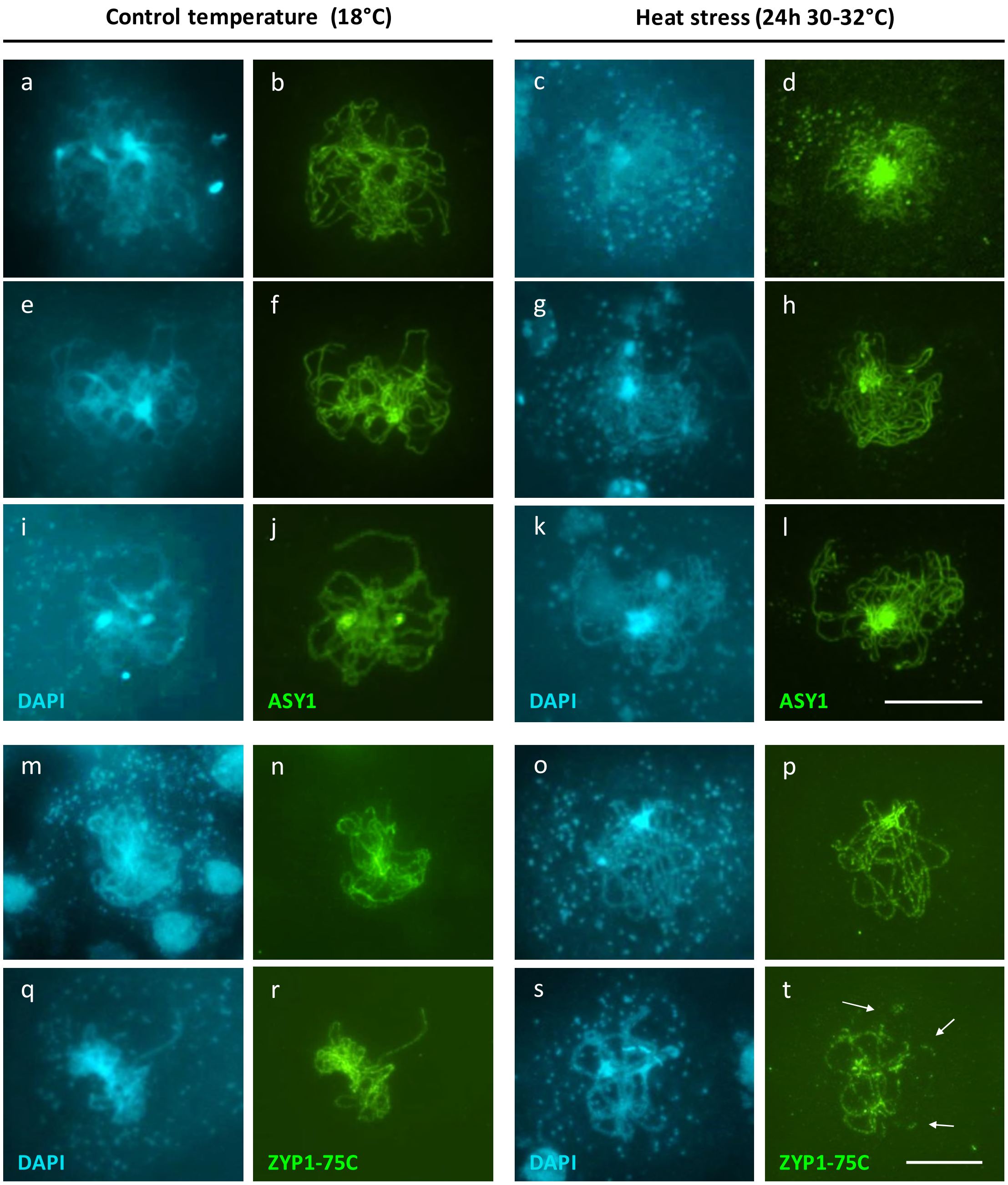

Supplementary Fig. 5. Immunolabeling of the synaptonemal proteins ASY1 and ZYP1 in male meiosis of Arabidopsis under normal temperature conditions (18°C) and upon heat (24h 30-32°C). a-l; Localization of the axis-related protein ASY1 in prophase I of Arabidopsis male meiosis under normal temperature (a, b, e, f, i and j) and upon heat stress (c, d, g, h, k and l). Chromosomes are labeled using the DNA stain DAPI (blue color; a, c, e, g, i and k) and ASY1 is detected using immunostaining with the corresponding antibody (green color; b, d, f, h, j and l). m-t; Localization of the transverse synaptonemal protein ZYP1 in prophase I of Arabidopsis male meiosis under normal temperature conditions (m, n, q and r) and upon heat stress (o, p, s and t). Chromosomes are labeled using DAPI (blue color; m, o, q and s) and ZYP1 is detected using immunostaining with the ZYP1-75C antibody (green color; n, p, r and t).

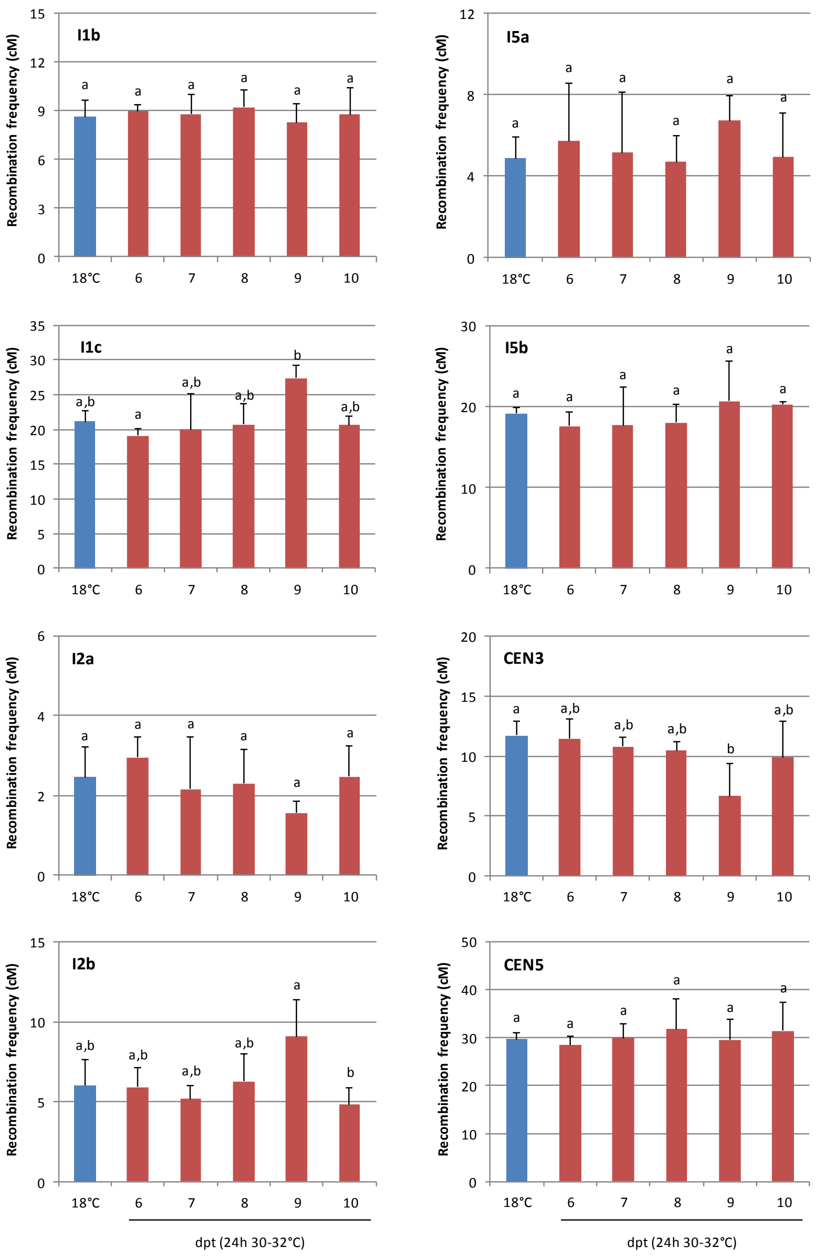

Supplementary Fig. 6. Histograms showing the male meiotic recombination frequency in different genomic regions, i.e. as measured based on the segregation of FTL fluorescent markers in mature pollen tetrads, in Arabidopsis *qrt1-2*$^{-/-}$ plants grown under normal temperatures (18-20°C, blue bars) and at different time periods following heat stress exposure (24h at 30-32°C, red bars). Dpt indicates ‘days post heat stress treatment’. Statistical differences in the recombination frequency were analysed using one-way ANOVA (Tukey HSD posthoc, α = 0,05) for normally distributed data or using the non-parametric Kruskal-Wallis test (α = 0,05) for data that are not normally distributed, and are indicated by letters above the corresponding bar plots.

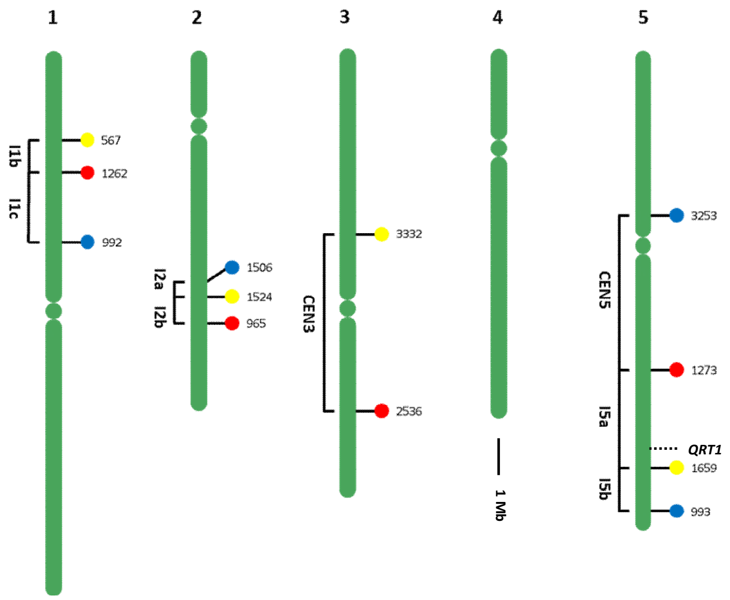

Supplementary Fig. 7. Schematic overview of the genomic location of the different FTL pollen reporters used for quantification of local CO frequency in male meiosis together with their corresponding interval, encoded fluorochrome (eCFP, YFP and dsRed) and individual number. The genomic position of the QRT1 gene on chromosome 5 is also indicated.

Supplementary Table 1. Quantitative analysis of the segregation of FTL markers in *qrt1-2*$^{-/-}$ pollen dyads and triads generated by heat stress (24h 30-32°C) to determine the level of FDR/SDR restitution. For each FTL reporter analysed, the frequency of FDR- and SDR-type configurations is presented for both dyads and triads, as well as for the combined pool of meiotically restituted figures. In addition, for each FTL reporter, the corresponding fluorochrome and FTL interval is listed, as well as its exact genomic position (Pos) on the chromosome (Chr) and distance from the centromere (Cen_dist). The total number of dyads and/or triads assessed for each FTL reporter are indicated by the value n.

FTL pollen-specific reporter						Dyads (%)			Triads (%)			Dyads and triads (%)		
Name	Reporter	Chr	Pos (bp)	Cen_dist (bp)	Interval	(n)	FDR	SDR	(n)	FDR	SDR	(n)	FDR	SDR
FTL_3332	YFP	III	11 115 724	1 384 276	CEN3	130	21,3	78,7	135	26,7	73,3	265	24,0	76,0
FTL_2536	DsRed2	III	16 520 560	3 020 560	CEN3	130	25,2	74,8	135	31,1	68,9	265	28,2	71,9
FTL_3253	AmCyan	V	9 304 032	3 195 968	CEN5	23	36,0	64,0	112	36,4	63,6	135	36,2	63,8
FTL_1273	DsRed2	V	18 164 269	5 664 269	CEN5	23	43,2	56,8	112	53,9	46,1	253	58,7	38,8
					I5ab	24	72,1	27,9	94	65,7	24,3			
FTL_1659	YFP	V	23 080 567	10 580 567	I5ab	24	96,4	3,6	94	74,7	25,3	118	85,6	14,5
FTL_993	CFP	V	25 731 311	13 231 311	I5ab	24	80,7	19,3	94	65,5	24,5	118	73,1	21,9

Supplementary Table 2. Impact of heat stress on male meiotic recombination frequency. Recombination frequency in different genomic intervals in Arabidopsis male meiosis, as determined by the segregation of two linked FTL markers in *qrt1-2*$^{-/-}$ tetrads at the mature pollen stage at normal temperature (18°C) as well as at various days following heat stress exposure (24h 30-32°C). FTL pollen analysed at 9 dpt result from male meiocytes that have been exposed to the heat stress treatment. Total number of mature pollen analysed is indicated by value (n), wheres the number of biological repeats is represented by value (r).

	I1b				I1c				I2a				I2b			
	(n)	(r)	Rec. Freq. (%)		(n)	(r)	Rec. Freq. (%)		(n)	(r)	Rec. Freq. (%)		(n)	(r)	Rec. Freq. (%)	
dpt			Mean	St. dev.			Mean	St. dev.			Mean	St. dev.			Mean	St. dev.
0	602	3	8,6	*1,04*	602	3	21,1	*1,70*	1394	6	2,5	*0,76*	1394	6	6,0	*1,58*
6	716	3	9,0	*0,39*	716	3	19,1	*1,00*	758	3	2,9	*0,50*	758	3	5,9	*1,26*
7	886	4	8,8	*1,19*	886	4	20,0	*5,23*	822	3	2,2	*1,32*	822	3	5,2	*0,80*
8	681	3	9,2	*1,08*	681	3	20,7	*2,92*	638	3	2,3	*0,85*	638	3	6,3	*1,69*
9	772	3	8,3	*1,12*	772	3	27,4	*1,79*	555	3	1,6	*0,29*	555	3	9,1	*2,29*
10	652	3	8,7	*1,64*	652	3	20,6	*1,22*	904	3	2,5	*0,78*	904	3	4,8	*1,01*

	I5a				I5b				CEN3				CEN5			
	(n)	(r)	Rec. Freq. (%)		(n)	(r)	Rec. Freq. (%)		(n)	(r)	Rec. Freq. (%)		(n)	(r)	Rec. Freq. (%)	
dpt			Mean	St. dev.			Mean	St. dev.			Mean	St. dev.			Mean	St. dev.
0	1043	6	4,9	*1,05*	1043	6	19,2	*0,76*	1423	6	11,7	*1,18*	1312	4	29,6	*1,37*
6	418	3	5,7	*2,83*	418	3	17,7	*1,72*	745	3	11,4	*1,64*	755	3	28,4	*1,76*
7	615	3	5,2	*2,98*	615	3	17,7	*4,72*	546	3	10,8	*0,80*	951	4	29,9	*3,01*
8	869	3	4,7	*1,27*	869	3	18,0	*2,26*	571	3	10,4	*0,77*	725	4	31,8	*6,41*
9	852	4	6,7	*1,20*	852	4	20,7	*4,98*	320	5	6,7	*2,77*	825	4	29,5	*4,24*
10	729	3	4,9	*2,18*	729	3	20,3	*0,35*	1438	7	9,9	*2,99*	828	3	31,4	*6,00*
