# Supplementary material for: High temperatures alter cross-over distribution and induce male meiotic restitution in Arabidopsis thaliana
Source: Commun Biol. 2020 Apr 23;3:187. doi: 10.1038/s42003-020-0897-1 (PMC7181631; doi:10.1038/s42003-020-0897-1)
Supplement: Supplementary file 3 — Reporting Summary [file 42003_2020_897_MOESM3_ESM.pdf]

nature research

Corresponding author(s): Danny Geelen
Last updated by author(s): Dec 16, 2019

Reporting Summary

Nature Research wishes to improve the reproducibility of the work that we publish. This form provides structure for consistency and transparency in reporting. For further information on Nature Research policies, see Authors & Referees and the Editorial Policy Checklist.

Statistics

For all statistical analyses, confirm that the following items are present in the figure legend, table legend, main text, or Methods section.

n/a	Confirmed	
☐	☒	The exact sample size (*n*) for each experimental group/condition, given as a discrete number and unit of measurement
☐	☒	A statement on whether measurements were taken from distinct samples or whether the same sample was measured repeatedly
☐	☒	The statistical test(s) used AND whether they are one- or two-sided<br>*Only common tests should be described solely by name; describe more complex techniques in the Methods section.*
☐	☐	A description of all covariates tested
☐	☐	A description of any assumptions or corrections, such as tests of normality and adjustment for multiple comparisons
☐	☒	A full description of the statistical parameters including central tendency (e.g. means) or other basic estimates (e.g. regression coefficient) AND variation (e.g. standard deviation) or associated estimates of uncertainty (e.g. confidence intervals)
☐	☒	For null hypothesis testing, the test statistic (e.g. *F*, *t*, *r*) with confidence intervals, effect sizes, degrees of freedom and *P* value noted<br>*Give P values as exact values whenever suitable.*
☐	☐	For Bayesian analysis, information on the choice of priors and Markov chain Monte Carlo settings
☐	☐	For hierarchical and complex designs, identification of the appropriate level for tests and full reporting of outcomes
☐	☐	Estimates of effect sizes (e.g. Cohen's *d*, Pearson's *r*), indicating how they were calculated

Our web collection on statistics for biologists contains articles on many of the points above.

Software and code

Policy information about availability of computer code

Data collection	Image data were collected using an Olympus IX81 inverted<br>670 fluorescence microscope equipped with an X-Cite 120 LED Boost lamp. Images were captured using an<br>671 Olympus XM10 camera. Bi-fluorescent images and Z-stacks were processed using ImageJ.
Data analysis	Statistical analysis of data and comparison of means was executed using the SPSS v25 software.

For manuscripts utilizing custom algorithms or software that are central to the research but not yet described in published literature, software must be made available to editors/reviewers. We strongly encourage code deposition in a community repository (e.g. GitHub). See the Nature Research guidelines for submitting code & software for further information.

Data

Policy information about availability of data

All manuscripts must include a data availability statement. This statement should provide the following information, where applicable:

- Accession codes, unique identifiers, or web links for publicly available datasets
- A list of figures that have associated raw data
- A description of any restrictions on data availability

All images are stored at a servers hosted by DICT unit of Ghent University. The raw data are stored as excel files. The data are stored in a folder named Nico De Storme.

Field-specific reporting

Please select the one below that is the best fit for your research. If you are not sure, read the appropriate sections before making your selection.

☒ Life sciences ☐ Behavioural & social sciences ☐ Ecological, evolutionary & environmental sciences

For a reference copy of the document with all sections, see nature.com/documents/nr-reporting-summary-flat.pdf

Life sciences study design

All studies must disclose on these points even when the disclosure is negative.

Sample size	We analyzed previously the frequency of dyad formation at regular temperature. About 0,5% pollen is diploid under normal temperature. We therefore analysed at least 200 spores to dyad minimally 1 dyad. Under stress conditions this figure increases to 5- 20%.<br>To determine the recombination frequency we measured at least 500 events. The frequency depends on the genetic distance. In regular conditions a marker set at the top and bottom has a chance of recombining 1 per meiosis.
Data exclusions	We did not exclude any data points
Replication	We repeated experiments at least three times (experiments separated in time). Similar results were obtained and averages were calculated.
Randomization	Randomization was not implemented in this study. Every heat stress experiment included a non-treatment control for which we analysed the same number of pollen as for the treated plants.
Blinding	Investigators were not blinded and new about the treatment of the analysed samples.

Reporting for specific materials, systems and methods

We require information from authors about some types of materials, experimental systems and methods used in many studies. Here, indicate whether each material, system or method listed is relevant to your study. If you are not sure if a list item applies to your research, read the appropriate section before selecting a response.

Materials & experimental systems

n/a	Involved in the study	
☐	☒	Antibodies
☒	☐	Eukaryotic cell lines
☒	☐	Palaeontology
☒	☐	Animals and other organisms
☒	☐	Human research participants
☒	☐	Clinical data

Methods

n/a	Involved in the study	
☒	☐	ChIP-seq
☒	☐	Flow cytometry
☒	☐	MRI-based neuroimaging

Antibodies

Antibodies used	Antibodies agains ASY1 and SYP were obtained from Christ Franklin. Secondary antibodies were from commercial sources as indicated in the materials and methods
Validation	Validation was based on previous uses by our colleagues and by us, showing an expected labeling pattern in plants grown under normal conditions
